# Risk factors, survival analysis, and nomograms for distant metastasis in patients with primary pulmonary large cell neuroendocrine carcinoma: A population-based study

**DOI:** 10.3389/fendo.2022.973091

**Published:** 2022-10-17

**Authors:** Zhuo Song, Lijuan Zou

**Affiliations:** Department of Radiation Oncology, The Second Affiliated Hospital, Dalian Medical University, Dalian, China

**Keywords:** Large cell neuroendocrine carcinoma (LCNEC), the Surveillance, Epidemiology, and End Results (SEER) database, nomogram, distant metastasis (DM), predictive model

## Abstract

**Introduction:**

Pulmonary large cell neuroendocrine carcinoma (LCNEC) is a rapidly progressive and easily metastatic high-grade lung cancer, with a poor prognosis when distant metastasis (DM) occurs. The aim of our study was to explore risk factors associated with DM in LCNEC patients and to perform survival analysis and to develop a novel nomogram-based predictive model for screening risk populations in clinical practice.

**Methods:**

The study cohort was derived from the Surveillance, Epidemiology, and End Results database, from which we selected patients with LCNEC between 2004 to 2015 and formed a diagnostic cohort (n = 959) and a prognostic cohort (n = 272). The risk and prognostic factors of DM were screened by univariate and multivariate analyses using logistic and Cox regressions, respectively. Then, we established diagnostic and prognostic nomograms using the data in the training group and validated the accuracy of the nomograms in the validation group. The diagnostic nomogram was evaluated using receiver operating characteristic curves, decision curve analysis curves, and the GiViTI calibration belt. The prognostic nomogram was evaluated using receiver operating characteristic curves, the concordance index, the calibration curve, and decision curve analysis curves. In addition, high- and low-risk groups were classified according to the prognostic monogram formula, and Kaplan–Meier survival analysis was performed.

**Results:**

In the diagnostic cohort, LCNEC close to bronchus, with higher tumor size, and with higher N stage indicated higher likelihood of DM. In the prognostic cohort (patients with LCNEC and DM), men with higher N stage, no surgery, and no chemotherapy had poorer overall survival. Patients in the high-risk group had significantly lower median overall survival than the low-risk group.

**Conclusion:**

Two novel established nomograms performed well in predicting DM in patients with LCNEC and in evaluating their prognosis. These nomograms could be used in clinical practice for screening of risk populations and treatment planning.

## Introduction

In 1991, the first report of pulmonary large cell neuroendocrine carcinoma (LCNEC) occurred. (1) In 2015, LCNEC was removed from the pathological classification of the large cell carcinomas and placed under pulmonary neuroendocrine neoplasms (NENs), which was revised by the World Health Organization and carried over to the latest 2021 edition. (2, 3) LCNEC is an uncommon pathologic type that accounts for 3% of all lung malignancies. (4) Recent reports indicated that its incidence increased year by year, from 0.01/100,000 people in 1990 to 1.8/100,000 people in 2010, with its annual mortality doubling between 2004 and 2015. The survival time and rates for patients with stage I–III LCNEC were close to those of patients with non-small cell lung cancer (NSCLC), whereas those of patients with stage IV were more like those of patients with small cell lung cancer (SCLC). (4, 5) In NENs, LCNEC is similar to SCLC and is a high-grade, rapidly progressing, easily metastatic malignancy. (6) The incidence of brain metastases in patients with LCNEC was significantly higher than in patients with SCLC or NSCLC. (4, 7) Sex, age, primary tumor site, TNM stage, surgery status, and chemotherapy have been shown to be independent risk factors for the prognosis of LCNEC in previous studies. (8–12) However, there is still controversy around the clinical management and treatment of LCNEC, such as using radiotherapy, and there are no standardized treatment approaches, especially for patients with LCNEC and distant metastasis (DM). As a result, a novel clinical predictive model is needed to assess the risk variables for incidence and prognosis of LCNEC with DM so that early intervention may be provided to this high-risk population.

Nomograms have been widely utilized in the prognostic analysis of cancer because of its capacity to graphically and intuitively show risk factors related to prognosis. (13, 14) Moreover, it has been used to assess metastasis in patients with osteosarcoma, with good results. (15) Therefore, we used the Surveillance, Epidemiology, and End Results (SEER) database to access public data and to evaluate the risk variables related to DM in patients with *de novo* (primary) LCNEC and to conduct further prognostic analysis. We present here two nomograms that can be used.

## Materials and methods

### Study population

We obtained data from SEER∗Stat software v8.3.9.2, released on 20 August 2021. (16) Data were extracted from the sub-database “Incidence–SEER Research Plus Data, 18 Registries, Nov 2020 Sub (2000–2018)”. Due to the limitations of the SEER database, the years of diagnosis were limited to 2004–2015 to ensure consistency in TNM staging. Then, according to the International Classification of Diseases for Oncology, Third Edition (ICD-O-3)/World Health Organization 2008, “Lung and Bronchus” was selected as the primary tumor site. Based on histologic type (ICD-O-3: 8013/3, 3762) patients with “LCNEC” were selected, and their original data were downloaded. Further data filtering was then performed in R software (version 4.1.2). (17) The exclusion criteria included (1): multiple primary tumors (2), age < 18 years, and (3) pathology grade I or II (low-grade), as the LCNEC is a high-grade neuroendocrine carcinoma. In addition, relevant clinicopathological characteristic, including age, sex, race, primary tumor site, laterality, pathology grade, TNM stage, and tumor size were required to be available. We enrolled 959 patients in the diagnostic cohort (**Supplementary Figure 1A**) and further excluded (1): patients with no DM and (2) survival time < 1 month, and (3) patients for whom data on surgery, chemotherapy, or radiotherapy status were not included. Then, 272 patients were enrolled in the prognostic cohort (**Supplementary Figure 1B**).

The study population was randomly split into training (70%) and validation groups (30%) in the diagnostic cohort, with a 7:3 ratio. The training and validation groups of the prognostic cohort were derived from the diagnostic cohort without regrouping. In each cohort, to investigate the factors associated with the incidence and the prognosis of DM in LCNEC, we created two nomogram models and performed a survival analysis. Both models were constructed using the training groups and validated using the validation groups.

### Variables collected

As part of the diagnostic cohort, the following variables were assessed: sex, age, race, laterality, T stage, N stage, primary site, pathological grade, and tumor size. In the prognostic cohort, variables included those in the diagnostic cohort as well as surgery, chemotherapy, and radiotherapy status. Further subgroup analysis was performed in the diagnostic and prognostic cohorts to prepare for nomogram establishment. Meanwhile, survival analysis was conducted in the prognostic cohort, with overall survival (OS) as the primary endpoint; OS was defined as the period from the initial diagnosis of LCNEC and death of any cause.

### Statistical analysis

The study cohort was randomly grouped to form a training and a validation group. All variables were reclassified as categorical variables, and the clinicopathological characteristics of LCNEC patients were compared using the Chi-squared test in the training and validation groups, some using Fisher’s exact test.

In the diagnostic cohort, we utilized logistic regression analysis to analyze risk variables of DM in patients with LCNEC patients. First, the univariate analysis was performed, with a two-sided P < 0.05 regarded as statistically significant, to identify risk factors. Then, significant variables were incorporated into the multivariable risk model, and odds ratios (ORs) and 95% confidence intervals (CIs) were computed. The independent risk factors selected by the model were then incorporated into a nomogram for visualization and clinical predictive analysis. Finally, we compared the novel nomogram with each individual risk variable using the receiver operating characteristic (ROC) curves of the training and validation groups and computed the area under the curve (AUC) to assess the validity of the novel nomogram. Decision curve analysis (DCA) and GiViTI calibration belt were used to assess the reliability of the nomogram.

In the prognostic cohort, the risk variables for OS in patients with LCNEC with DM were assessed using Cox proportional hazards regression analysis. The variables with statistical significance (2-sided P < 0.05) from the univariate analysis were applied to the multivariable analysis to screen individual risk factors related to prognosis. Hazard ratios (HRs) and 95% CIs were also calculated at the same time. According to the results of the univariate and multivariable analyses, a prognostic nomogram was established. The validity of the nomogram was assessed using the concordance index (C-index), as well as time-dependent ROC curves at 1, 2, and 3 years, based on the nomogram and individual prognostic risk factors. The reliability of the nomogram was evaluated using DCA curves and the calibration curves at 1, 2, and 3 years. All validations were carried out in the training group and the validation group. In addition, the nomogram algorithm was used to determine the individual risk score of risk variables. Based on the median risk score, the prognostic cohort was separated into high- and low-risk groups to prepare for the survival analysis. The Kaplan–Meier method was applied to assess the OS of the two risk groups, and the log-rank test was used to obtain P-values in the training and validation groups.

R software and associated packages, including “table1”, “regplot”, “pROC”, “ROCR”, “givitiR”, “rms”, “ggDCA”, “survival”, “survminer”, and “survivalROC” were used for the aforementioned statistical analyses.

## Results

### Baseline characteristics of the diagnostic and prognostic cohorts

Our study included two major study cohorts, the diagnostic cohort of patients with LCNEC and the prognostic cohort of patients with LCNEC with DM. Baseline characteristics are presented in **Tables 1** and **2**. Among the 959 patients with LCNEC patients, patients were most commonly elderly and male. The most common primary tumor site was in the lung, and the most common tumor size was ≤3 cm. Among the tumor stages, T2 and N0 were the most common, and 308 patients had DM (M1). In the diagnostic cohort (**Table 1**), all patients with LCNEC were randomized into a training and a validation group. The Chi-squared test (some using Fisher’s exact test) revealed no significant differences in any of the covariates between the two groups, indicating that the grouping was completely random. The training and validation groups had mean ages of 64.93 years (range, 18–92 years; interquartile range, 58–72 years) and 65.62 years (range, 45–90 years; interquartile range, 59–73 years), respectively. In the prognostic cohort (**Table 2**), the grouping was entirely consistent with the diagnostic cohort. There were 272 patients with LCNEC with DM, most of them were elderly men as before. The most common tumor stages were T4 and N2. The primary tumor site was still common in the lung. For treatment, most patients received chemotherapy and radiotherapy, but few underwent surgery.

**Table 1:** Baseline characteristics of pulmonary large cell neuroendocrine carcinoma (LCNEC) patients (in the diagnostic cohort).

	Training (N=671)	Validation (N=288)	Overall (N=959)	χ2	P
Sex				2.523	0.112
Female	321 (47.8%)	121 (42.0%)	442 (46.1%)		
Male	350 (52.2%)	167 (58.0%)	517 (53.9%)		
Age, years				0.498	0.919
≥18 and <60	194 (28.9%)	83 (28.8%)	277 (28.9%)		
≥60 and <70	247 (36.8%)	101 (35.1%)	348 (36.3%)		
≥70 and <80	181 (27.0%)	80 (27.8%)	261 (27.2%)		
≥80	49 (7.3%)	24 (8.3%)	73 (7.6%)		
Race				3.523	0.172
Black	85 (12.7%)	26 (9.0%)	111 (11.6%)		
Other	26 (3.9%)	8 (2.8%)	34 (3.5%)		
White	560 (83.5%)	254 (88.2%)	814 (84.9%)		
Laterality					0.374
Left	300 (44.7%)	126 (43.8%)	426 (44.4%)		
Right	371 (55.3%)	161 (55.9%)	532 (55.5%)		
Bilateral	0 (0%)	1 (0.3%)	1 (0.1%)		
Primary site					0.770
Lung	640 (95.4%)	272 (94.4%)	912 (95.1%)		
Bronchus	23 (3.4%)	12 (4.2%)	35 (3.6%)		
Overlapping lesion of lung	8 (1.2%)	4 (1.4%)	12 (1.3%)		
Grade				0.677	0.411
III	506 (75.4%)	225 (78.1%)	731 (76.2%)		
IV	165 (24.6%)	63 (21.9%)	228 (23.8%)		
T				1.041	0.791
T1	195 (29.1%)	80 (27.8%)	275 (28.7%)		
T2	275 (41.0%)	114 (39.6%)	389 (40.6%)		
T3	41 (6.1%)	22 (7.6%)	63 (6.6%)		
T4	160 (23.8%)	72 (25.0%)	232 (24.2%)		
N				0.996	0.802
N0	347 (51.7%)	158 (54.9%)	505 (52.7%)		
N1	87 (13.0%)	36 (12.5%)	123 (12.8%)		
N2	184 (27.4%)	71 (24.7%)	255 (26.6%)		
N3	53 (7.9%)	23 (8.0%)	76 (7.9%)		
M				1.114	0.291
M0	448 (66.8%)	203 (70.5%)	651 (67.9%)		
M1	223 (33.2%)	85 (29.5%)	308 (32.1%)		
Tumor size, cm				1.190	0.755
≤3	285 (42.5%)	122 (42.4%)	407 (42.4%)		
>3 and ≤5	176 (26.2%)	84 (29.2%)	260 (27.1%)		
>5 and ≤7	107 (15.9%)	41 (14.2%)	148 (15.4%)		
>7	103 (15.4%)	41 (14.2%)	144 (15.0%)		

**Table 2:** Baseline characteristics of pulmonary large cell neuroendocrine carcinoma (LCNEC) patients with distant metastasis (in the prognostic cohort).

	Training (N=196)	Validation (N=76)	Overall (N=272)	χ2	P
Sex				2.008	0.157
Female	90 (45.9%)	27 (35.5%)	117 (43.0%)		
Male	106 (54.1%)	49 (64.5%)	155 (57.0%)		
Age, years				2.116	0.549
≥18 and <60	60 (30.6%)	22 (28.9%)	82 (30.1%)		
≥60 and <70	72 (36.7%)	30 (39.5%)	102 (37.5%)		
≥70 and <80	47 (24.0%)	21 (27.6%)	68 (25.0%)		
≥80	17 (8.7%)	3 (3.9%)	20 (7.4%)		
Race					0.584
Black	25 (12.8%)	12 (15.8%)	37 (13.6%)		
Other	7 (3.6%)	1 (1.3%)	8 (2.9%)		
White	164 (83.7%)	63 (82.9%)	227 (83.5%)		
Laterality					0.043
Left	79 (40.3%)	39 (51.3%)	118 (43.4%)		
Right	117 (59.7%)	36 (47.4%)	153 (56.3%)		
Bilateral	0 (0%)	1 (1.3%)	1 (0.4%)		
Primary site					0.804
Lung	175 (89.3%)	67 (88.2%)	242 (89.0%)		
Bronchus	18 (9.2%)	7 (9.2%)	25 (9.2%)		
Overlapping lesion of lung	3 (1.5%)	2 (2.6%)	5 (1.8%)		
Grade				0.194	0.660
III	148 (75.5%)	60 (78.9%)	208 (76.5%)		
IV	48 (24.5%)	16 (21.1%)	64 (23.5%)		
T					0.728
T1	25 (12.8%)	10 (13.2%)	35 (12.9%)		
T2	83 (42.3%)	27 (35.5%)	110 (40.4%)		
T3	9 (4.6%)	3 (3.9%)	12 (4.4%)		
T4	79 (40.3%)	36 (47.4%)	115 (42.3%)		
N				0.537	0.911
N0	53 (27.0%)	23 (30.3%)	76 (27.9%)		
N1	25 (12.8%)	10 (13.2%)	35 (12.9%)		
N2	84 (42.9%)	29 (38.2%)	113 (41.5%)		
N3	34 (17.3%)	14 (18.4%)	48 (17.6%)		
Tumor size, cm				0.236	0.972
≤3	51 (26.0%)	18 (23.7%)	69 (25.4%)		
>3 and ≤5	55 (28.1%)	23 (30.3%)	78 (28.7%)		
>5 and ≤7	40 (20.4%)	15 (19.7%)	55 (20.2%)		
>7	50 (25.5%)	20 (26.3%)	70 (25.7%)		
Surgery				0.036	0.849
No	164 (83.7%)	65 (85.5%)	229 (84.2%)		
Yes	32 (16.3%)	11 (14.5%)	43 (15.8%)		
Chemotherapy				0.091	0.763
No	59 (30.1%)	25 (32.9%)	84 (30.9%)		
Yes	137 (69.9%)	51 (67.1%)	188 (69.1%)		
Radiotherapy				0.030	0.863
No	84 (42.9%)	31 (40.8%)	115 (42.3%)		
Yes	112 (57.1%)	45 (59.2%)	157 (57.7%)		

### Diagnostic predictive model of DM in patients with LCNEC

**Figure 1:**
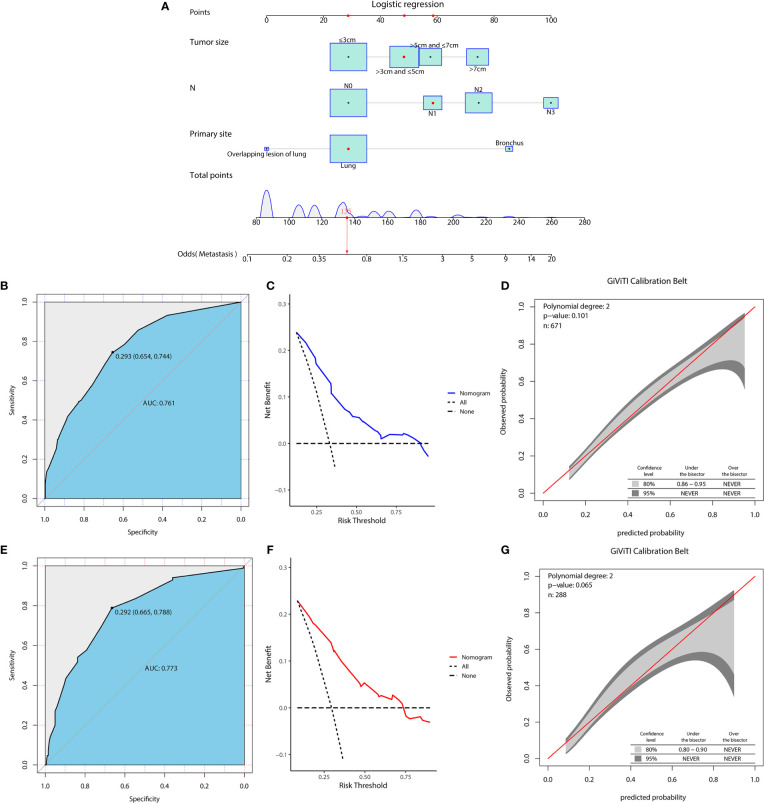
A diagnostic nomogram was developed for predicting the risk of distant metastasis in patients with LCNEC **(A)**. The receiver operating characteristic (ROC) curve **(B)**, decision curve analysis (DCA) curve **(C)**, and the GiViTI calibration belt **(D)** of the training group, and the ROC curve **(E)**, DCA curve **(F)**, and the GiViTI calibration belt **(G)** of the validation group were used to evaluate the validity and reliability of the nomogram.

**Figure 2:**
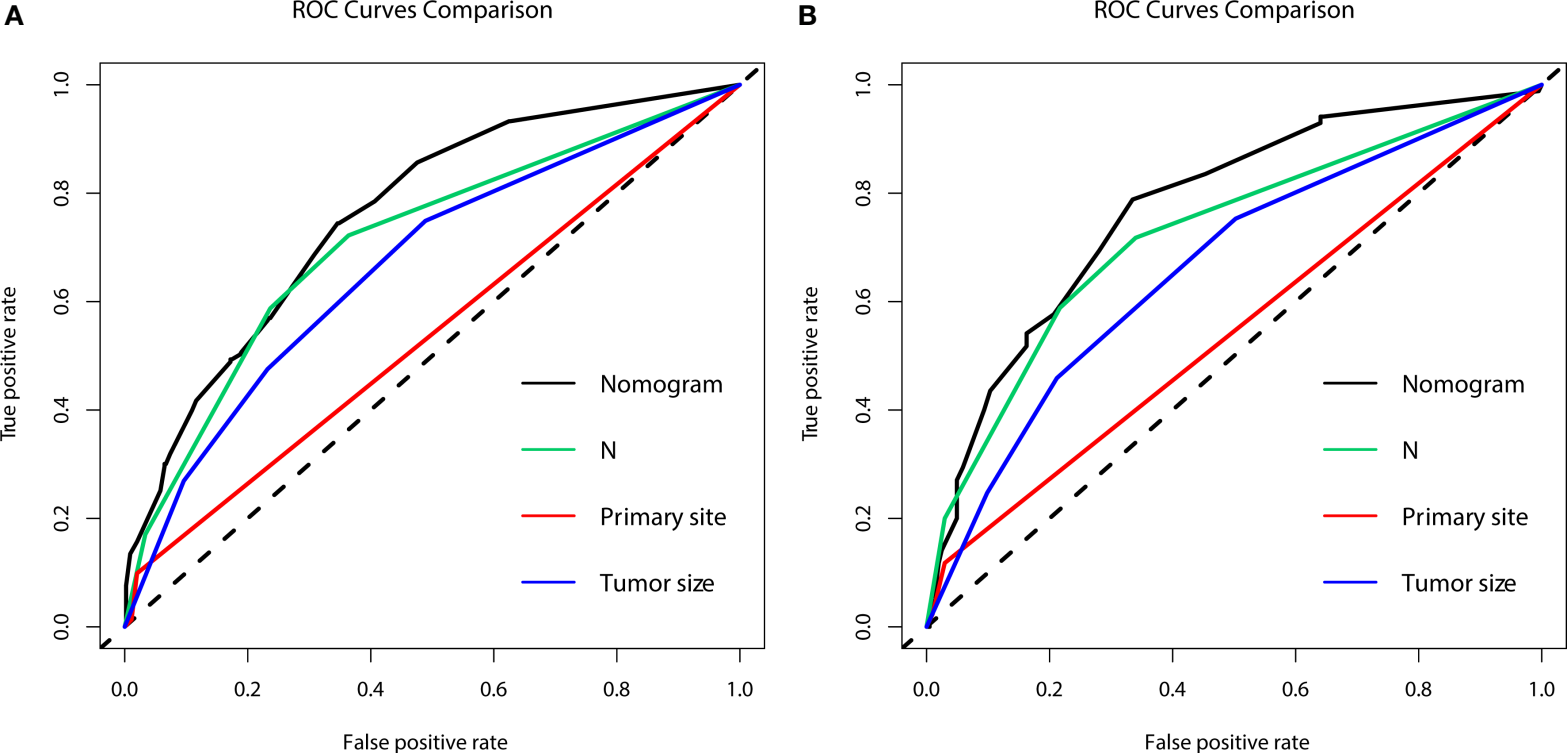
The area under the receiver operating characteristic curves (AUCs) were compared for the diagnostic nomogram in the training group **(A)** and validation group **(B)** with all independent variables, including N stage, primary site, and tumor size.

In the diagnostic cohort, the results of logistic regression analysis are shown in **Table 3**. First, a univariate analysis found five variables that may be associated with DM in LCNEC, including tumor size, primary tumor site, T stage, N stage, and sex. However, we excluded the T stage as it could be contradictory to clinical practice. These variables were then further incorporated into a multivariable analysis, which ultimately revealed three independent risk factors associated with DM, namely, a primary site of LCNEC closer to bronchus, and patients were more likely to have DM with larger tumor size and higher N stage. Additionally, to access the risk of DM, three independent risk variables were combined into a novel diagnostic predictive model, and a nomogram was generated in the training group (**Figure 1A**). Then, the ROC curves were drawn, with AUCs of 0.761 and 0.773 for the training and validation groups, respectively (**Figures 1B, E**). DCA both in the training and validation groups (**Figures 1C, F**) demonstrated the reliability of the nomogram. Moreover, we plotted the GiViTI calibration belts, which showed that the 95% CI did not cross the diagonal bisector at 45 degrees, and the P-values for the training and validation groups were 0.101 and 0.065, respectively (**Figures 1D, G**), indicating that the nomogram was reliable for predicting DM. (18) Meanwhile, for each individual risk factor, ROC curves were created, and the diagnostic nomogram outperformed any single factor in the training and validation groups (**Figures 2A, B**).

**Table 3:** Analyses of distant metastasis in LCNEC patients using univariate and multivariate logistic regression.

	Univariate analysis			Multivariate analysis		
	OR	95%CI	P	OR	95%CI	P
Sex						
Female	Reference			Reference		
Male	1.336	1.062-1.683	0.038	1.102	0.850 -1.430	0.537
Age, years						
≥18 and <60	Reference					
≥60 and <70	0.897	0.677-1.189	0.526			
≥70 and <80	0.817	0.602-1.107	0.274			
≥80	0.938	0.588-1.477	0.820			
Race						
Black	Reference					
Other	0.740	0.359-1.465	0.478			
White	0.824	0.584-1.172	0.359			
Laterality						
Left	Reference					
Right	1.071	0.851-1.348	0.624			
Bilateral	NA	NA	0.967			
Primary site						
Lung	Reference			Reference		
Bronchus	7.777	4.100 -15.948	<0.001	3.192	1.582 -6.914	0.009
Overlapping lesion of lung	1.646	0.597 -4.312	0.398	0.925	0.303 -2.714	0.906
Grade						
III	Reference					
IV	0.918	0.699-1.199	0.600			
T						
T1	Reference					
T2	2.653	1.910-3.729	<0.001			
T3	1.679	0.930-2.938	0.137			
T4	7.893	5.561-11.345	<0.001			
N						
N0	Reference			Reference		
N1	2.436	1.677-3.519	<0.001	2.206	1.497 -3.231	<0.001
N2	4.759	3.590-6.332	<0.001	3.864	2.882 -5.194	<0.001
N3	12.760	8.102-20.575	<0.001	8.754	5.452 -14.350	<0.001
Tumor size, cm						
≤3	Reference			Reference		
>3 and ≤5	2.118	1.569-2.864	<0.001	1.759	1.274-2.429	0.004
>5 and ≤7	3.265	2.317-4.607	<0.001	2.313	1.592-3.358	<0.001
>7	5.510	3.908-7.811	<0.001	3.588	2.456-5.258	<0.001

NA, Not applicable.

### Prognostic predictive model of patients with LCNEC with DM

**Figure 3:**
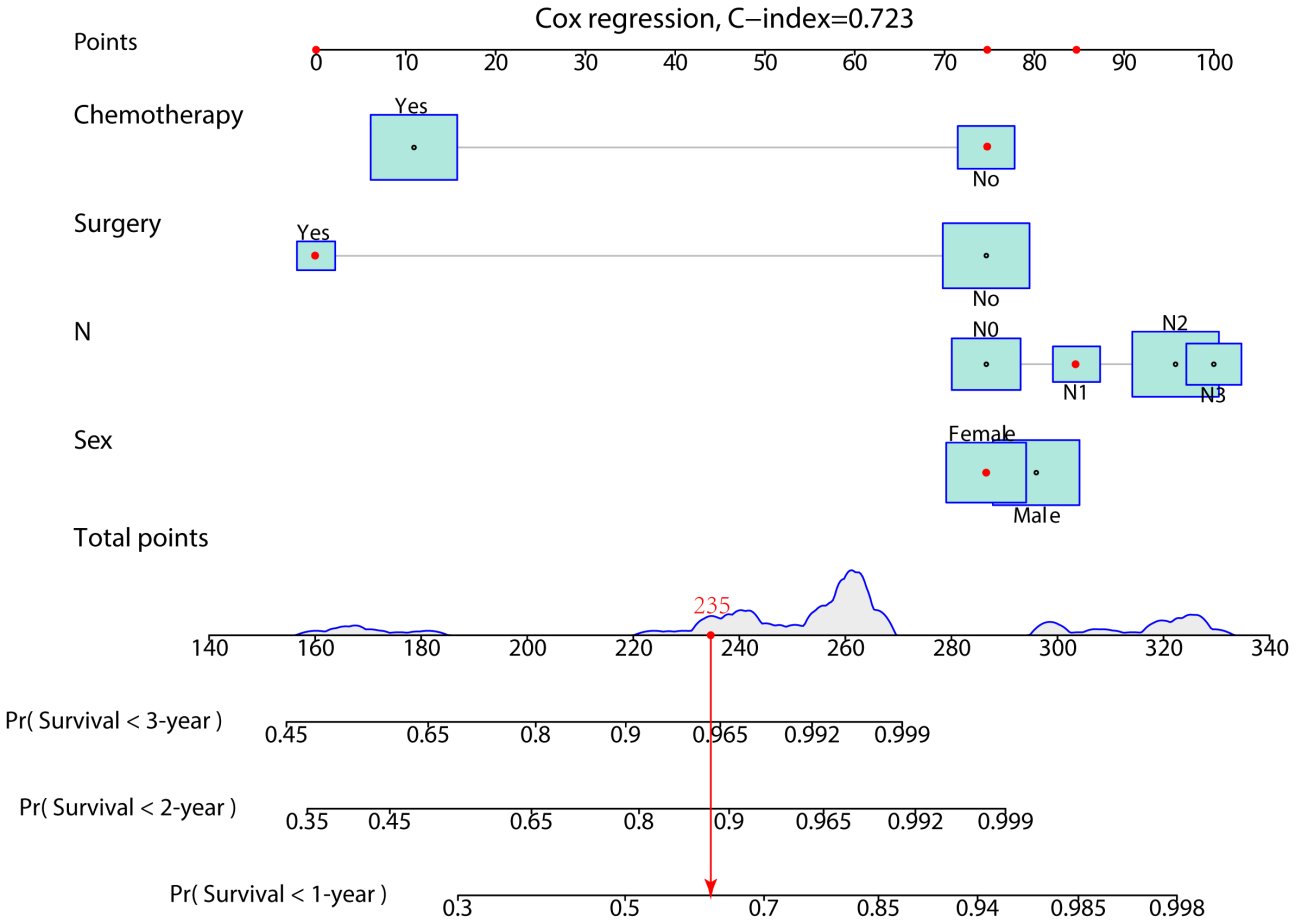
A prognostic nomogram was developed for predicting the 1-, 2-, and 3-year OS of patients with LCNEC with distant metastasis.

**Figure 4:**
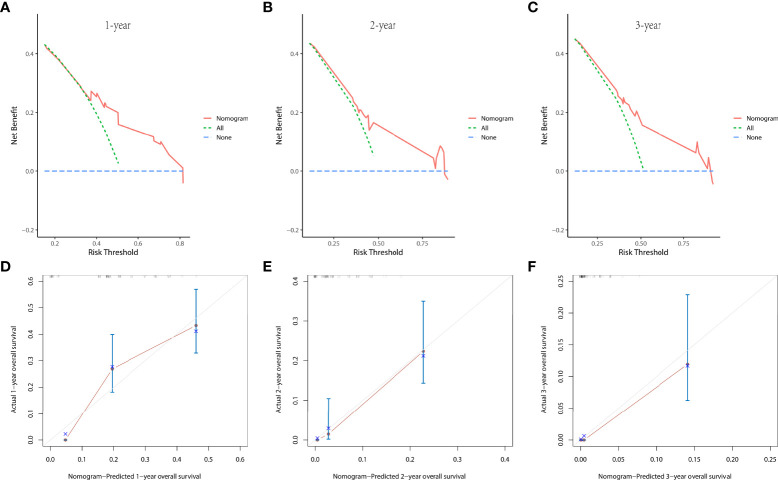
The decision curve analysis (DCA) curves at 1 **(A)**, 2 **(B)**, and 3 years **(C)** and the calibration curves at 1 **(D)**, 2 **(E)**, and 3 years **(F)** in the training group were used to evaluate the reliability of the prognostic nomogram.

**Figure 5:**
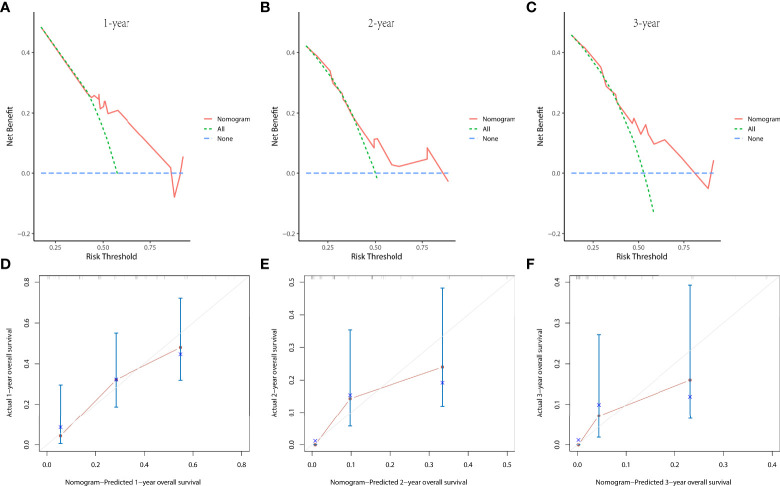
The decision curve analysis (DCA) curves at 1 **(A)**, 2 **(B)**, and 3 years **(C)** and the calibration curves at 1 **(D)**, 2 **(E)**, and 3 years **(F)** in the validation group were used to evaluate the reliability of the prognostic nomogram.

**Figure 6:**
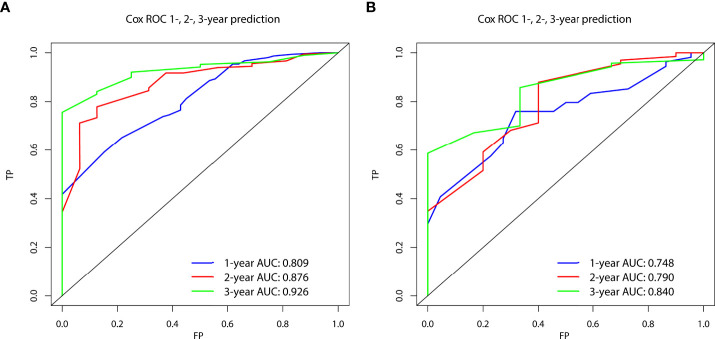
The time-dependent receiver operating characteristic (ROC) curves at 1, 2, and 3 years in the training group **(A)** and in the validation group **(B)** were used to evaluate the validity of the prognostic nomogram.

**Figure 7:**
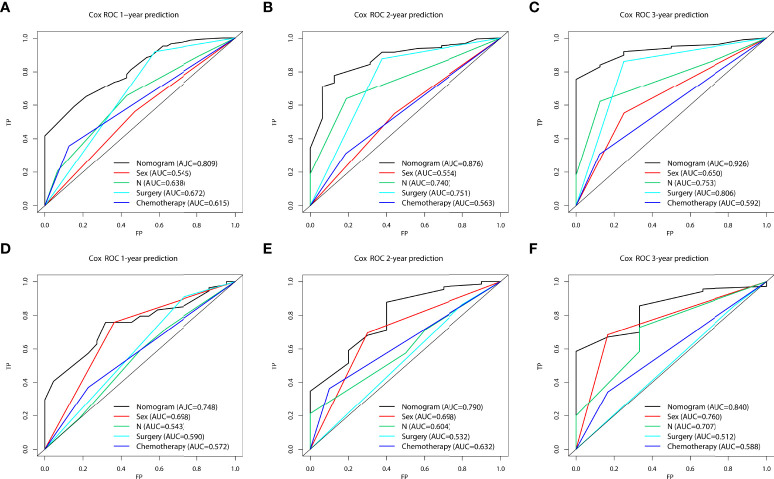
The area under the receiver operating characteristic curves (AUCs) were compared for the prognostic nomogram in the training and validation groups with all independent variables, including Sex, N stage, Surgery, and Chemotherapy at 1 **(A, D)**, 2 **(B, E)**, and 3 years **(C, F)**.

In the prognostic cohort, univariate and multivariable Cox proportional hazards regression analyses were performed to search for factors linked with OS in patients with LCNEC with DM (**Table 4**). Four variables were selected, including sex, N stage, surgery, and chemotherapy status. Specifically, male sex, no surgery, no chemotherapy, and a higher N stage were independent risk factors, highly associated with worse OS. Then, in the training group, we created a prognostic nomogram based on these four risk variables (**Figure 3**) and validated it in the validation group. First, in patients with LCNEC with DM, the nomogram could be utilized to predict OS at 1, 2, and 3 years. In the training and validation groups, the appropriate DCA (**Figures 4A–C**, **5A–C**) and calibration curves (**Figures 4D–F**, **5D–F**) are shown. These results suggested the prognostic nomogram was a feasible predictive model. Second, the time-dependent ROC curves at 1, 2, and 3 years of the nomogram proved the model performed well in prognostic analysis, with respective AUCs of 0.809, 0.876, and 0.926 in the training group (**Figure 6A**) and 0.748, 0.790, and 0.840 in the validation group (**Figure 6B**). Together with a concordance index of 0.723, on the one hand, these results confirmed the validity of the prognostic nomogram, and, on the other hand, the nomogram seemed to be better at predicting long-term survival. Additionally, the ROC curves of the prognostic nomogram were compared to those of all individual risk variables, and it was shown that the prognostic nomogram outperformed any single factor at 1, 2, and 3 years in the training (**Figures 7A–C**) and validation groups (**Figures 7D–F**).

**Table 4:** Analyses of overall survival in LCNEC patients with distant metastasis using univariate and multivariate Cox regression.

	Univariate analysis				Multivariate analysis		
	HR		95%CI	P	HR	95%CI	P
Sex							
Female	Reference				Reference		
Male	1.427		1.158-1.757	0.005	1.468	1.179-1.828	0.004
Age, years							
≥18 and <60	Reference				Reference		
≥60 and <70	1.277		0.994-1.640	0.109	1.134	0.876-1.467	0.423
≥70 and <80	1.407		1.067-1.856	0.042	1.346	1.014-1.788	0.085
≥80	2.272		1.488-3.469	0.001	1.451	0.934-2.254	0.165
Race							
Black	Reference						
Other	0.936		0.491-1.785	0.866			
White	1.172		0.869-1.581	0.384			
Laterality							
Left	Reference						
Right	1.278		1.039-1.572	0.051			
Bilateral	NA		NA	0.205			
Primary site							
Lung	Reference				Reference		
Bronchus	1.546		1.090-2.194	0.041	1.550	1.066-2.256	0.054
Overlapping lesion of lung	1.808		0.855-3.823	0.193	2.379	1.113-5.082	0.060
Grade							
III	Reference						
IV	1.084		0.854-1.376	0.579			
T							
T1	Reference						
T2	1.129		0.813-1.569	0.542			
T3	1.066		0.600-1.893	0.855			
T4	1.855		1.332-2.583	0.002			
N							
N0	Reference				Reference		
N1	1.085		0.769-1.531	0.696	1.181	0.827-1.688	0.442
N2	1.591		1.237-2.045	0.002	1.479	1.138-1.921	0.014
N3	1.857		1.351-2.552	0.001	1.730	1.231-2.432	0.008
Tumor size, cm							
≤3		Reference					
>3 and ≤5		1.272	0.964-1.679	0.154			
>5 and ≤7		1.268	0.938-1.713	0.195			
>7		1.687	1.265-2.251	0.003			
Surgery							
No		Reference			Reference		
Yes		0.424	0.318-0.566	<0.001	0.320	0.229-0.445	<0.001
Chemotherapy							
No		Reference			Reference		
Yes		0.499	0.400-0.621	<0.001	0.292	0.226-0.378	<0.001
Radiotherapy							
No		Reference					
Yes		1.016	0.828-1.247	0.899			

NA, Not applicable.

### Outcomes of survival analysis

**Figure 8:**
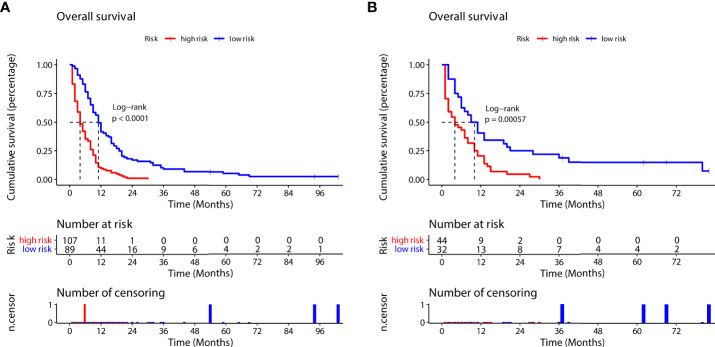
Survival outcomes in the training group **(A)** and validation group **(B)** for the high-risk and low-risk groups (according to the prognostic nomogram formula).

According to the prognostic nomogram, we then utilized the Kaplan–Meier method to evaluate the OS of both the high- and low-risk groups. Median survival time in the high- group and low-risk groups was 4 and 11 months, respectively, in the training group (**Figure 8A**), and 4 months and 10 months, respectively, in the validation group (**Figure 8B**). Compared to the low-risk group, the high-risk group had significantly lower OS (training group, p<0.0001; validation group, p=0.00057).

## Discussion

Pulmonary LCNEC shows a high prevalence of lymph node metastases (60-80%) and DM (40%) at the time of diagnosis, with a median survival time for individuals with pulmonary LCNEC who develop DM of about five months. (19, 20) Therefore, we must take effective measures to diagnose DM of LCNEC as early as possible to provide appropriate treatment time. In the present study, to screen for high-risk groups, we developed two nomograms for the diagnostic and prognosis analysis of patients with LCNEC with DM and categorized them according to the risk score produced by the model. First, the larger the primary tumor and the closer the tumor to bronchus, the more likely it was to metastasize. Second, the prognosis of patients with LCNEC who had DM was improved by surgery and chemotherapy, but it was worse in male patients than in female patients. Third, regional lymph node metastasis was a significant risk factor affecting the prognosis of patients with LCNEC, which was related to the occurrence of DM and the prognosis of patients with LCNEC patients with DM.

### Diagnostic cohort

Recently, studies focusing on clinical characteristics and prognosis of LCNEC have been published. Lowczak et al. showed that LCNEC, as with SCLC, was frequently associated with male sex, heavy smoking, and advanced age (median age of 65 years). (21) Cao et al. indicated that, although fewer older individuals with pulmonary LCNEC underwent surgery, chemotherapy, or radiation therapy, aggressive and effective treatment could increase survival time dramatically. (22) This corresponds with our study, which showed patients with LCNEC were more commonly older men. However, because the SEER database lacks smoking information, we did not evaluate the relationship between smoking and DM. Interestingly, in the present study, although in the prognostic cohort, the majority of LCNEC patients with DM are still elders (≥60 years old, nearly 70%), age was not a risk factor for DM; this requires further study to validate. In addition, the results in the T3 staging seem to be contrary to clinical practice. On the one hand, since the TNM staging in this study was the 6th edition staging, this may be due to deficiencies in the staging itself; an updated staging system may solve this problem, suggesting that it may be better to evaluate whether a patient with LCNEC is susceptible to DM based on tumor size rather than T staging. Additionally, since our study focused on patients with LCNEC with DM, the patients with an advanced tumor stage may have been more similar to SCLC in terms of features and prognosis. However, recent studies have shown that not all LCNEC harbors the neuroendocrine profile of SCLC, implying that some LCNECs have features of NSCLC, especially molecular features. (23) Rekhtman et al. found commonly genomic alterations in LCNEC including the genes for p53 (TP53; 78%), retinoblastoma (RB1; 38%), serine/threonine kinase 11 (STK11; 33%), kelch-like ECH associated protein 1 (Keap1; 31%) and the Kirsten rat sarcoma viral oncogene (KRAS) (22%). In addition, NSCLC-like LCNEC exhibited more frequent mutations in NOTCH family genes (28%), which may be key regulators of neuroendocrine differentiation. (24) Accordingly, LCNEC could be divided into two major subsets, one with SCLC-like mutations, including the biallelic inactivation of tumor protein RB1 and TP53, and the other with NSCLC-like mutations, including biallelic inactivation of KEAP1/STK11. (25) A refined classification of LCNEC will influence diagnosis, prognosis, and treatment decisions, (26) and may be useful in assessing the presence of DM.

### Prognostic cohort

In our study, sex was not a factor associated with the development of DM in patients with LCNEC, but was a factor affecting the prognosis of those with DM. The prognosis of male patients was worse than that of women. Recent studies found that lifestyle, tobacco use, secondhand smoke exposure, several occupational exposures, treatment type received, duration of anticancer treatment after diagnosis, endogenous circulating levels of sex hormones, and expression and mutation rates of several related genes (including EGFR, KRAS, and P53) had differences between men and women, and that sex differences have important implications for lung cancer development, prognosis, and treatment preferences. (27, 28) In addition, one immunohistochemistry marker, the Ki-67 proliferation index (PI), may have an effect on the prognosis of LCNEC, and recent studies have shown that Ki-67 PI≥55% was strongly associated with poor survival. (29, 30) Hermans et al. showed that patients with stage IV LCNEC with a solitary brain metastasis and N0/N1 disease more commonly had a Ki67 PI ≤ 40%, and these patients had better prognosis than those with Ki67 PI>40%. (31) However, Walts et al. suggested that a blanket use of 20%, 40%, or any other Ki-67 cut-off to diagnose LCNEC or analyze prognosis was inaccurate. (32) Unfortunately, the lack of Ki-67 data in the SEER database prevented further exploration in the present study, and it is hoped that large multicenter studies will be available to assess this.

Regarding treatment modalities, although previous studies have explored the treatment of LCNEC, the results were limited, contradictory, and rare for patients with LCNEC with DM. In our analyses, to investigate the positive effects of surgery and chemotherapy on patients with LCNEC with DM, we used multivariate Cox regression analysis and survival analysis, but, due to limited information in the SEER database, we were unable to conduct further analysis. The main findings of previous studies are as follows. First, primary surgical treatment significantly improved survival in patients with LCNEC patients, even in those with stage IV. (10) However, LCNEC had a high postoperative recurrence rate, with more than half relapsing within one year, although the R0 resection margin and N0 status (no lymph node metastasis) improved the time to recurrence. (33) As a result, even for LCNEC patients with an earlier stage, surgery alone was insufficient. (34) Second, chemotherapy alone could be more beneficial than other treatments, even for patients in stage IV. (8) The best treatment approaches are still being explored. Fisch et al. suggested that aggressive systemic therapy for metastatic LCNEC, including platinum doublets and immunotherapy, could improve OS. (35) Genomic, such as cell free DNA analysis and next-generation sequencing, subtyping was helpful for therapeutic decision-making and prognostication of patients with LCNEC. (36, 37) However, Hadoux et al. found that, in patients with LCNEC receiving platinum–etoposide chemotherapy, retinoblastoma protein (Rb) status had no influence on prognosis. (38) Therefore, the relationship between gene expression and treatment regimens requires further study. Third, there is still controversy about radiotherapy. On the one hand, radiotherapy could prolong the survival of patients with LCNEC, including those in stage IV, especially those who have received chemotherapy or have not undergone surgery. (39) However, radiotherapy may shorten the survival time of individuals undergoing surgery. (40–42) On the other hand, it is interesting to note that the metastatic pattern of LCNEC is similar to NSCLC, but the prognosis is similar to that of SCLC. (43)The brain was the most common metastatic site, so prophylactic cranial irradiation is an effective treatment and might be improve survival time. (44) In patients with LCNEC with brain metastases, stereotactic radiosurgery is superior to whole brain radiation treatment. (45) Moreover, Girelli et al. reported that patients with LCNEC with lymph node metastasis had a poor prognosis, and more active multidisciplinary approaches were needed. (46) Overall, surgery combined with chemotherapy may be an appropriate treatment for LCNEC with DM, especially in patients with regional lymph node metastasis.

### Advantages and shortcomings

Previous studies on patients with LCNEC with DM were limited, and most of them were single-center studies with a lack of validation. The advantages of the present study are that the data came from the SEER database, the sample size was large, and the follow-up period was long. We created an entirely new nomogram for visualization, to predict independent risk factors for the occurrence and prognosis of DM in individuals with LCNEC, that could be used for screening high-risk patients and guiding personalized treatment in clinical practice.

Nevertheless, there are some shortcomings in the present study. First, the number of patients with LCNEC with DM was only 272, and as this was a retrospective study, this may have led to potential bias. Second, although our nomograms have been internally validated in both the training and validation groups, more data is needed to determine the wider applicability of the external validation model. Third, there is a lack of key information in the SEER database that may be relevant to survival, for example, smoking history, performance status, tumor biomarkers status, genetic testing results, specific treatment modality; these data can help further refine our model. In particular, the recent increase in use of immunotherapy and targeted therapy in lung cancer may offer new hope for patients with LCNEC with DM. Kim et al. showed that the PD-1/PD-L1 pathway was found to be activated in the LCNEC microenvironment and associated with a high mutation burden. (47) Vrontis et al. suggested that treatment and management of patients with advanced LCNEC could be achieved with SCLC approaches, such as platinum–etoposide–atezolizumab chemotherapy, which can improve prognosis. (48) Additional prospective randomized controlled studies are needed.

## Conclusions

In the present cohort study, individual risk variables and prognostic factors for DM in patients with LCNEC were identified using two regression analysis approaches and related variables were applied to establish a new predictive model and perform further survival analysis. Meanwhile, two novel nomograms were developed, including a diagnostic nomogram and a prognostic nomogram, and these could be reliable tools for clinical screening of risk populations and for optimizing treatment.

## Data availability statement

Publicly available datasets were analyzed in this study. This data can be found here: Surveillance, Epidemiology, and End Results (SEER) Program. SEER*Stat Software version 8.3.9.2. https://seer.cancer.gov/seerstat/download. Accessed 12 Mar 2022.

## Ethics statement

This study was exempt from the approval processes of the Institutional Review Boards because the SEER database patient information is de-identified. Therefore, a patient consent form was not applicable.

## Author contributions

All authors contributed to the study conception and design. Data collection and analysis were performed by ZS. The first draft of the manuscript was written by ZS and all authors commented on previous versions of the manuscript. All authors read and approved the final manuscript.

## Acknowledgments

The authors are grateful to the open access SEER database and R software for providing us with the raw research data and for the data analysis, and to all the professors and friends who have helped us with the use of related software. This manuscript was edited for English Language by Charlesworth Author Services (www.cwauthors.com).

## Conflict of interest

The authors declare that the research was conducted in the absence of any commercial or financial relationships that could be construed as a potential conflict of interest.

## Publisher’s note

All claims expressed in this article are solely those of the authors and do not necessarily represent those of their affiliated organizations, or those of the publisher, the editors and the reviewers. Any product that may be evaluated in this article, or claim that may be made by its manufacturer, is not guaranteed or endorsed by the publisher.

## Supplementary material

Supplementary Figure 1 Flow diagram for the diagnostic cohort **(A)** and the prognostic cohort **(B)**.
