# Supplementary figures and images for: Risk factors, survival analysis, and nomograms for distant metastasis in patients with primary pulmonary large cell neuroendocrine carcinoma: A population-based study
Source: Front Endocrinol (Lausanne). 2022 Oct 17;13:973091. doi: 10.3389/fendo.2022.973091 (PMC9623680; doi:10.3389/fendo.2022.973091)

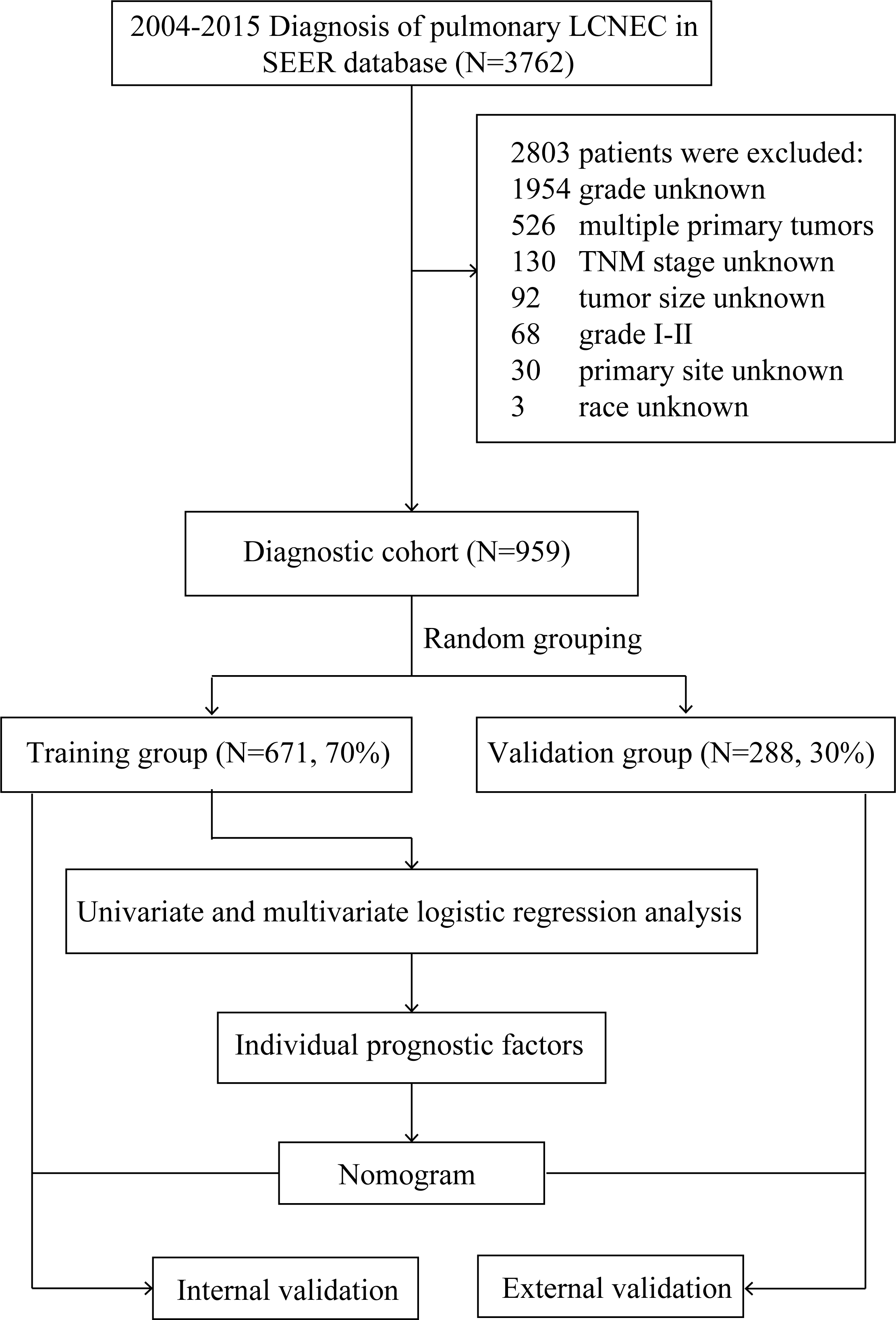

Supplement: Supplementary Figure 1 — Flow diagram for the diagnostic cohort (A) and the prognostic cohort (B). [file Image_1.tif]

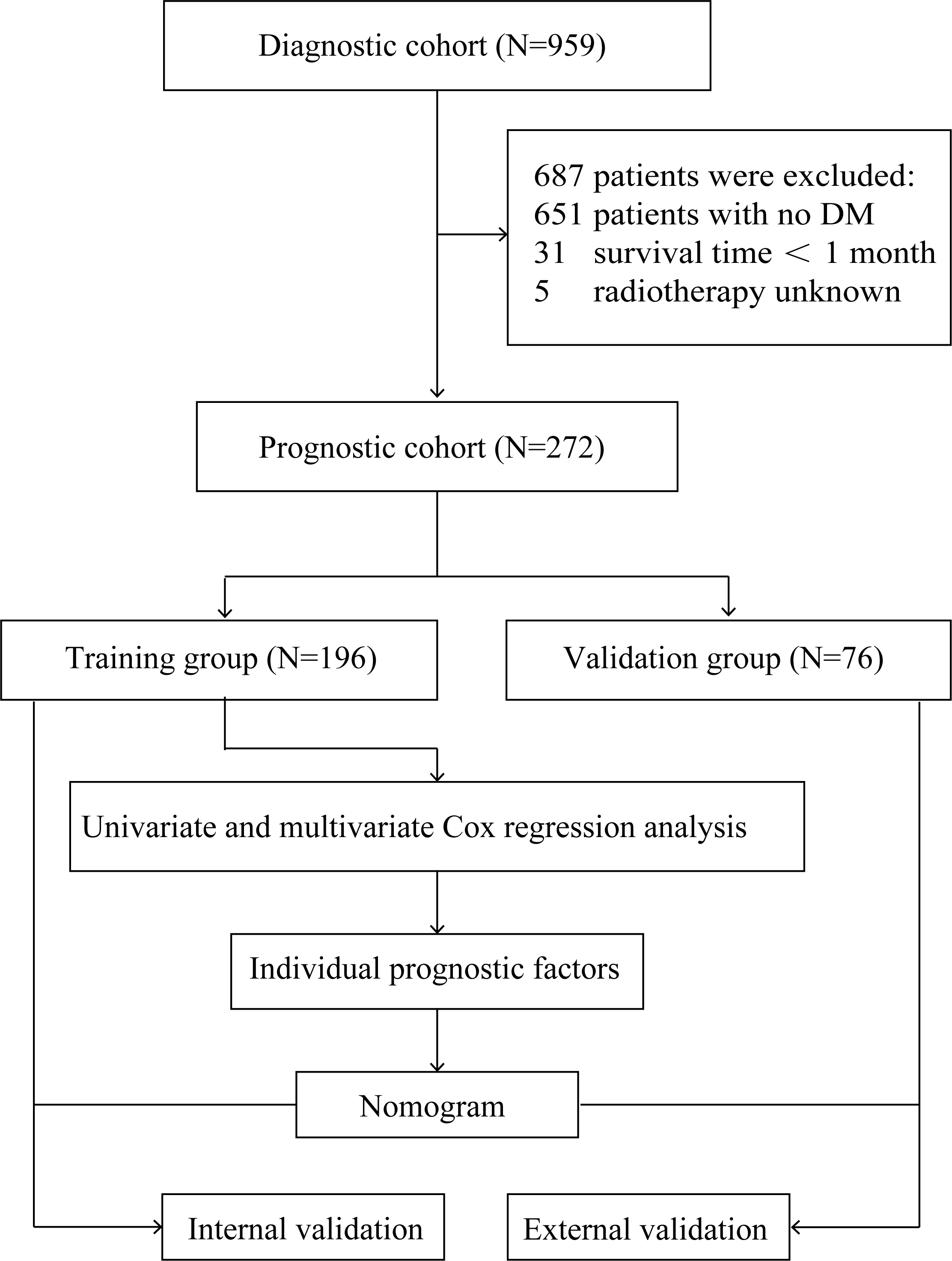

Supplement: Supplementary file 2 [file Image_2.tif]
